# Effect of high pH on growth of *Synechocystis* sp. PCC 6803 cultures and their contamination by golden algae (*Poterioochromonas* sp.)

**DOI:** 10.1007/s00253-015-7024-0

**Published:** 2015-11-06

**Authors:** Eleftherios Touloupakis, Bernardo Cicchi, Ana Margarita Silva Benavides, Giuseppe Torzillo

**Affiliations:** Istituto per lo Studio degli Ecosistemi, CNR, Via Madonna del Piano 10, I-50019 Sesto Fiorentino, Italy; Escuela de Biología, Universidad de Costa Rica, San Pedro, San José, 2060 Costa Rica; Centro de Investigación en Ciencias del Mar y Limnología (CIMAR), Universidad de Costa Rica, San Pedro, San José, 2060 Costa Rica

**Keywords:** *Synechocystis* PCC 6803, *Poterioochromonas* sp., Contamination, Fluorescence

## Abstract

Culturing cyanobacteria in a highly alkaline environment is a possible strategy for controlling contamination by other organisms. *Synechocystis* PCC 6803 cells were grown in continuous cultures to assess their growth performance at different pH values. Light conversion efficiency linearly decreased with the increase in pH and ranged between 12.5 % (PAR) at pH 7.5 (optimal) and decreased to 8.9 % at pH 11.0. Photosynthetic activity, assessed by measuring both chlorophyll fluorescence and photosynthesis rate, was not much affected going from pH 7.5 to 11.0, while productivity, growth yield, and biomass yield on light energy declined by 32, 28, and 26 % respectively at pH 11.0. Biochemical composition of the biomass did not change much within pH 7 and 10, while when grown at pH 11.0, carbohydrate content increased by 33 % while lipid content decreased by about the same amount. Protein content remained almost constant (average 65.8 % of dry weight). Cultures maintained at pH above 11.0 could grow free of contaminants (protozoa and other competing microalgae belonging to the species of *Poterioochromonas*).

**Electronic supplementary material:**

The online version of this article (doi:10.1007/s00253-015-7024-0) contains supplementary material, which is available to authorized users.

## Introduction

The cyanobacterium *Synechocystis* PCC 6803 is being widely used as a model organism for the study of photosynthetic processes, since it is well characterized and can easily be transformed. Moreover, its genome has already been completely sequenced, and a variety of mutants has become available. The use of *Synechocystis* PCC 6803 (hereafter *Synechocystis*) has been proposed for the production of biohydrogen as well as chemicals and biomaterials (Gao et al. 2012; Sharma et al. 2011; Yu et al. 2013; Englund et al. 2014). It has also been genetically engineered for the photosynthetic production of isoprene, a hydrocarbon currently used as feedstock in the synthetic chemistry industry for the production of commercial commodities (Chaves et al. 2015).

One of the major problems emerging in mass cultures is the lack of a reliable control of contamination by other microorganisms. Large-scale microalgae cultures like terrestrial crops can be attacked by pests and weeds causing devastating effects. Closed systems are usually recommended for strains growing in non-selective media. However, a number of recent reports have indicated that cultures in closed systems are often affected by contaminants in spite of their protection from the outside atmosphere (Rego et al. 2015; Hoffman et al. 2008; Forehead and O’Kelly 2013; Carney and Lane 2014; Zemke et al. 2013). Indeed, it has been found that in many cases, the main vehicle of the contamination is represented by the water used for preparing the medium.

One of the greatest dangers experienced by us in the mass cultivation of *Synechocystis* was represented by flagellates belonging to the species of *Poterioochromonas* (*Synurophyceae*). These are common members of the freshwater planktonic communities and possess digestive vacuoles in order to use phagotrophy to supplement phototrophic growth. They are very efficient phagotrophs, showing high growth rates, and have been identified as potential contaminants in *Synechocystis* mass cultures (Holen and Boraas 1995). Their growth is supported by a heterotrophic metabolism with cyanobacterial cells representing the feeding prey. In addition to the feeding mechanism, this kind of contamination has what can be defined a “killing effect” on the culture, as when these flagellates are phagotrophically active, they produce toxins with strong antibiotic effect (Leeper and Porter 1995; Blom and Pernthaler 2010).

It has been reported that, in order to prevent the growth of invading microorganisms in cyanobacteria cultures, the pH can be increased towards alkalinity since many cyanobacteria can still grow despite such harsh environmental conditions (Pikuta and Hoover 2007; McGinn et al. 2011). *Synechocystis* possesses a CO_2_-concentrating mechanism enabling them to acquire and concentrate inorganic carbon from the extracellular environment (Badger and Price 2003). Moreover, they can also utilize HCO_3_^−^ as carbon source, by converting it to CO_2_ with the enzyme carbonic anhydrase. Many mechanisms have been suggested for pH homeostasis and the regulation of CO_2_/HCO_3_ concentration, such as expression of proteins responsible for carbon assimilation and pH homeostasis, regulation of periplasmic carbonic anhydrase activity, or accumulation of acetolactate ions (Maestri and Joset 2000; Summerfield and Sherman 2008; Battchikova et al. 2010). Inorganic carbon availability is a key factor to consider when setting up a cyanobacterial cultivation at very alkaline pH since, at these pH values, inorganic carbon is mainly present as carbonate. Several experimental and modeling attempts have been made in order to elucidate how pH can affect cyanobacterial metabolism (Summerfield and Sherman 2008; Lopo et al. 2012).

In view of a potential utilization of *Synechocystis* in mass cultivation, and the necessity to prevent pollution of the culture by growing them at pH above the optimum, we wished to assess the effect of alkaline pH on productivity and on the level of contamination of the culture.

## Methods

### Preparation of inoculum

*Synechocystis* strain PCC 6803 cells (kindly provided by Prof. Tamagnini, IMI, Portugal) were pre-cultured in a BG11 medium under artificial irradiance of 50 μmol photons m^−2^ s^−1^, supplied from one side of the cultivation columns (i.d. = 50 mm; 400 mL working volume). The columns were placed in thermostatic bath at 28 °C and bubbled with a mixture of air-CO_2_ (97/3 *v*/*v*) at a continuous flow rate of 5 dm^3^ min^−1^.

### Continuous culture

A 1-L Pyrex Roux-type photobioreactor (PBR) with a flat cross section (12 × 5 cm width) and a flat bottom was used. The culture was illuminated using cool white lamps (Dulux L, 55W/840, Osram, Italy) with a fixed photon flux density (PFD) of 150 μmol photons m^−2^ s^−1^. Mixing of the culture was achieved by means of a specially designed rotating impeller driven magnetically by a stirrer at the bottom (Giannelli et al. 2009). The pH of the culture was maintained at the pre-set value by automatic addition of CO_2_, while the temperature was maintained at a constant value of 28.0 ± 0.2 °C. The cultures were operated according to a continuous culture regime (chemostat) by imposing a fixed dilution rate (*D*, h^−1^) of 0.036 h^−1^. The culture was assumed to be at a steady state when the dry weight (DW) of the culture remained unchanged for at least 36 h. The actual biomass yield on light energy was determined from the following equation: *Y*_kJ_ = (*D* × *V* × *X*)/(*A* × *I*_a_), where *D* is the dilution rate (0.036 h^−1^), *V* is the working volume (1 L), *X* is the cell concentration (g L^−1^), *A* is the area of the PBR exposed to light irradiation (190 cm^2^), and *I*_a_ is the intensity of light absorbed by the cells (in μmol photons cm^−2^ h^−1^).

### Analytical procedures

DW was determined in duplicate by using 10-mL samples taken from the culture daily. Samples were filtered through pre-weighted 47-mm-diameter glass microfiber membranes (Whatman GF/F filters, Maidstone, England). The cells were washed twice with deionized water and then oven-dried at 105 °C until constant weight. Chlorophyll concentration was determined spectrophotometrically in triplicate 5-mL samples which were centrifuged in glass tubes for 8 min at 2650*g* in an ALC-PK110 centrifuge. The pellet was re-suspended in 5 mL of pure methanol, placed in a 70 °C water bath for 3 min, and centrifuged again for 8 min at 2650*g*. The supernatant absorbance was measured at 665 and 750 nm against a pure methanol blind. The concentration of individual carotenoids was assessed using a reversed-phase Beckman System Gold HPLC (module 125 solvent) equipped with a diode array detector, model 168 Nouveau (Beckman Instruments, Inc., CA, USA), with a column Luna C8 (Phenomenex), in accordance with Van Heukelem and Thomas (2001). For phycobilisome measurements, culture samples (5 mL) were collected in tubes and centrifuged at 2650*g* for 8 min. The supernatant was discarded, and 0.5 mL of glass beads (diameter 0.17–0.18 mm, B. Braun Biotech Int, Germany) was added to the sample, along with 200 μL of NaCl 0.15 M phosphate-buffered (pH 7.4) solution. The mixture was vortexed for 10 min in order to break the cells; phosphate buffer was then added to reach a volume of 5 mL. The tubes were centrifuged at 2650*g* for 5 min, and the supernatant was then transferred into 15-mL Falcon tubes and centrifuged at 12,500*g* for 10 min.

The concentration of allophycocyanin (Apc) and phycocyanin (Pc) was calculated according to Bennett and Bogorad (1973).

Protein determination was performed in triplicate according to Lowry et al. (1951). Total carbohydrate content was measured using the phenol-sulfuric acid method (Dubois et al. 1956). Lipids were extracted from 5 mg of dry biomass using 1 mL of dichloromethane, 2 mL of methanol, and 0.8 mL of deionized water (1:2:0.8, *v*/*v*/*v*). The mixture was vortexed and sonicated for 10 min, after which an additional 1 mL of dichloromethane and 1 mL of deionized water were added. The mixture was then vortexed and centrifuged for 5 min at 1500*g* (ALC-PK110). The bottom phase was recovered, placed in pre-weighted containers, and heated to complete evaporation. The extracted lipids were then weighed. Amino acid composition was determined according to Potenza et al. (2013). DNA was extracted by using the DNeasy Blood and Tissue Kit (Qiagen) using the manufacturer’s protocol. DNA concentration was measured using the NanoDrop ND-1000 UV/Vis spectrophotometer according to the manufacturer’s instructions (NanoDrop Technologies, USA).

Elemental composition analysis of the biomass was performed on lyophilized samples using a CHNOS analyzer (Flash EA, 1112 Series, Thermo Electron Corporation). Ash content was determined after heating the biomass at 450 °C for 24 h. Lyophilized samples were analyzed for Ca, Mg, and Na concentrations by using an inductively coupled plasma emission spectrometer (PerkinElmer Optima 2000, Germany).

The heat of combustion (kJ g^−1^) of the biomass at the steady state was calculated by using the following formula: ([(proteins × 5.7) + (carbohydrates × 4.2) + (lipids × 9.3)]/100) × 4.184.

### Fluorescence measurements

Chlorophyll a fluorescence transients were recorded using a Handy PEA (Hansatech Instruments) in 2-mL dark-adapted samples illuminated with continuous light (650 nm peak wavelength, 3500 μmol photons m^−2^ s^−1^) provided by light-emitting diodes. Each chlorophyll a fluorescence induction curve was analyzed using “Biolyzer HP3” software. Analysis of chlorophyll fluorescence quenching was carried out with a pulse-amplitude-modulation fluorometer (PAM-2100, H. Walz, Effeltrich, Germany) operated by PC software PamWin (version 2.00f).

The ratio between variable and maximum fluorescence, *F*_v_/*F*_m_, was used to determine the maximum photochemical yield of photosystem II (PSII). For this purpose, samples were taken from the PBR and incubated in the dark for 15 min to remove any energy-dependent quenching. In addition, just before sending a flash for the *F*_m_ determination, a sample was illuminated with a 10-s-long far-red light pulse (above 700 nm, 10 W m^−2^), supplied by the PAM-2100. The effective photochemical quantum yield of PSII Δ*F*/*F*_m_′ = (*F*_m_′ − *F*_s_)/*F*_m_′, which is the number of electrons generated per photon absorbed, was measured using *F*_s_ and *F*_m_′, which represented the steady state and maximum fluorescence measured in the light. *F*_s_ and *F*_m_′ were measured in situ by pointing the fiber optic cable directly on the surface of the PBR and perpendicularly to the direction of the incident light.

Non-photochemical quenching (NPQ) was calculated by using the Stern-Volmer equation NPQ = (*F*_m_ − *F*_m_′)/*F*_m_′ (Krause and Janhns 2004). *F*_0_′ was estimated from the following relationship: *F*_0_′ = *F*_0_/(*F*_v_/*F*_m_ + *F*_0_/*F*_m_′). The photochemical quenching (qP) was calculated by using the Kooten and Snel equation (Kooten and Snel 1990).

The average chlorophyll-specific optical absorption cross section a* (normalized to chlorophyll *a* content, m^2^ mg chl^−1^) of the cells was determined according to Falkowski and Raven (1997).

### Oxygen evolution measurements

Oxygen evolution measurements were carried out in triplicate on 2-mL culture samples (chlorophyll content 5 mg L^−1^), using a Liquid-Phase Oxygen Electrode Chamber (Hansatech, DW3) thermostated at 28 °C and equipped with an oxygen control electrode unit (Hansatech, Oxy-lab). Light was supplied via a red LED light source (Hansatech LH36/2R) at a wavelength of 637 nm providing a 600-μmol photons m^−2^ s^−1^ PFD. The O_2_ concentration dissolved in the sample was continuously monitored at an acquisition rate of 0.2 reading s^−1^. Dark respiration rates were measured after the photosynthesis rates had been measured.

Preparation of predator

*Poterioochromonas* sp. strain ISE1 (CCALA1090; Supplementary Material Fig. S1) was from the Culture Collection of Autotrophic Organisms (CCALA). It was isolated from the central deionized water-producing plant of the Institute of Ecosystem Study (Florence, Italy) and grown in MWC medium (Guillard and Lorenzen 1972) .

### Grazing experiments

Cultures of *Synechocystis* were grown in 800 mL PBR and exposed to a PFD of 150 μmol m^−2^ s^−1^ and maintained at a constant temperature of 28 °C. Cultures were bubbled with air and subjected to a light-dark cycle (L = 10 h, D = 14 h). To assess the effect of pH on grazing capacity by *Poterioochromonas* sp., cultures of *Synechocystis* were contaminated with 1 % of *Poterioochromonas* cells and exposed to different pH conditions.

### Light microscopy

Cell suspensions mounted on microscope slides and covered were examined in bright field using a Nikon Eclipse E600. Microscopic fields were photographed using a Nikon digital Sight DS-UV1 digital camera. Digital slide photographs had brightness and contrast optimized to enhance regions of interest. Cell count was carried out using a Bürker-Türk counting chamber.

## Results

### Growth characterization of the culture

**Fig. 1:**
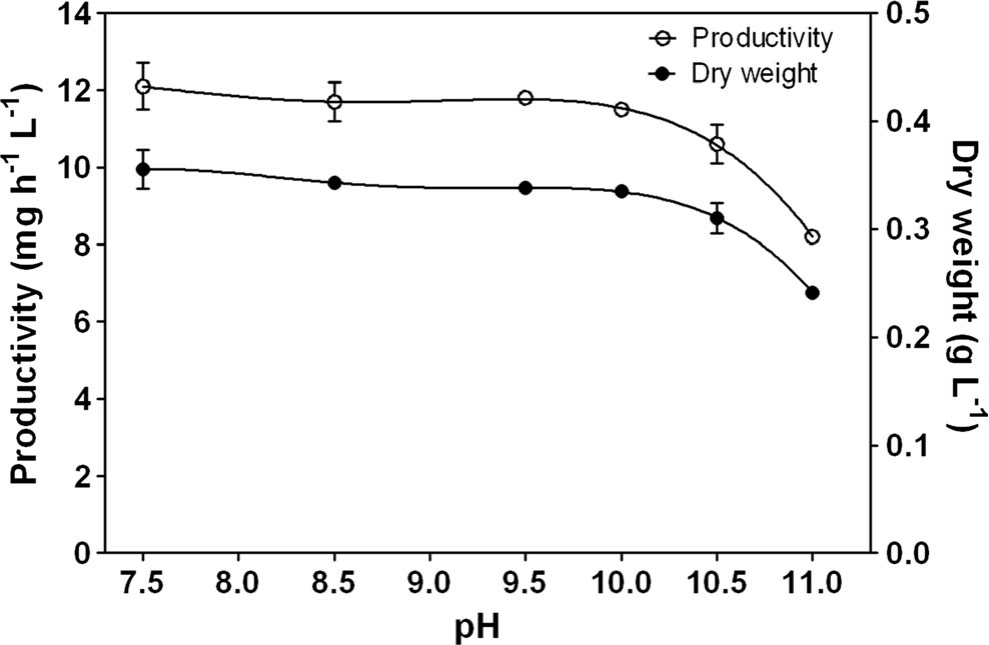
Changes in productivity and dry weight as a function of pH. Data are the average of at least three measurements; *error bars* represent the standard deviation

The relationship between pH and productivity is shown in Fig. 1. *Synechocystis* cultures grown at pH 7.5 showed an optimal productivity. Between pH 7.5 and 10, the reduction of productivity was scarce (5 %), while a further increase of the pH to 11.0 caused a drop of productivity of more 30 %. Being the dilution rate constant at 0.036 h^−1^, during the entire duration of the experiments, the reduction in productivity was the result of a corresponding reduction in the dry weight (Fig. 1).

Growth yield *Y*_kJ_ (the amount of dry biomass synthesized per kJ of light energy absorbed) reached a maximum value of 5.49 ± 0.27 mg kJ^−1^ at pH 7.5 and decreased at increasing pH (Table 1). As for the reduction in productivity, the drop between pH 10 and 11 was much more apparent. Multiplying the *Y*_kJ_ values by the heat of combustion of the biomass, calculated at each pH, it was possible to estimate the PAR-based light conversion efficiency (LCE) for each culture condition (Table 1). LCE ranged between 12.5 % (pH 7.5) and 8.9 % (pH 11.0), and the most significant drop was found moving from pH 10 (11.8 %) to pH 11 (8.9 %) (Table 1).

**Table 1:** Biomass dry weight, productivity, growth yield, actual biomass yield on light energy, and light conversion efficiency (LCE) of *Synechocystis* grown at different pH values. Values are mean ± standard deviations

pH	Dry weight (mg L^−1^)	Productivity (mg L^−1^ h^−1^)	*Y*_kJ_ (mg kJ^−1^)	*Y* (g mol photons^−1^)	LCE (%)
7.5	355 ± 18	12.1 ± 0.6	5.49 ± 0.27	1.20 ± 0.06	12.5 ± 0.6
8.5	343 ± 4	11.7 ± 0.5	5.31 ± 0.05	1.16 ± 0.01	11.9 ± 0.1
9.5	338 ± 3	11.8 ± 0.1	5.24 ± 0.05	1.15 ± 0.01	11.9 ± 0.6
10.0	335 ± 4	11.5 ± 0.1	5.20 ± 0.05	1.14 ± 0.01	11.8 ± 0.1
10.5	310 ± 14	10.6 ± 0.5	4.88 ± 0.22	1.07 ± 0.05	10.7 ± 0.4
11.0	241 ± 2	8.2 ± 0.1	3.94 ± 0.03	0.89 ± 0.01	8.9 ± 0.2

The actual biomass yield on light energy (*Y*), that is, the ability of photosynthetic microorganisms to utilize the light energy supplied for biomass formation, decreased as pH increased in the culture, and it ranged from 1.20 g biomass mol photons^−1^ at the optimal pH to 0.89 g mol photons^−1^ at the highest pH tested (Table 1).

### Fluorescence and photosynthetic parameters

The maximum quantum yield, calculated by the *F*_v_/*F*_m_ ratio, did not change much at the various pH values and was 0.48, indicating that *Synechocystis*’ PSII photochemistry was unaffected in the range of pH 7.5–11.0. NPQ values of chlorophyll fluorescence were found to be very low, and qP was consistently at relatively high values between 0.85 and 0.90 in the range of pH 7.5–10.5, which indicates that most of the absorbed energy was used for photochemistry (Table 2). A NPQ increase and a qP decrease appears at pH 11. The effective photochemical quantum yield of PSII (Δ*F*/*F*_m_′) remained stable at the range of 0.40–0.42 in all pH conditions except at pH 11.0 where it decreased by 11 % (Δ*F*/*F*_m_′ = 0.37) (Table 2).

**Table 2:** The effective photochemical quantum yield of PSII (Δ*F*/*F*_m_′), non-photochemical quenching (NPQ), photochemical quenching coefficient (qP), chlorophyll optical-absorption cross section (a*), O_2_ evolution, and respiration rates of *Synechocystis* cultured at different pH conditions. Values are mean ± standard deviations calculated over the steady state for each pH condition

pH	Δ*F*/*F*_m_′	NPQ	qP	a* (cm^2^ mg chl^−1^)	Net O_2_ evolution (μmol mg chl^−1^ h^−1^)	Respiration (μmol mg chl^−1^ h^−1^)
7.5	0.412 ± 0.007	0.011 ± 0.005	0.843 ± 0.004	105 ± 1	346 ± 33	22 ± 3
8.5	0.400 ± 0.007	0.066 ± 0.024	0.843 ± 0.001	110 ± 1	353 ± 1	19 ± 2
9.5	0.400 ± 0.001	0.054 ± 0.006	0.857 ± 0.010	109 ± 4	320 ± 4	22 ± 3
10.0	0.414 ± 0.007	0.075 ± 0.007	0.848 ± 0.008	109 ± 1	338 ± 7	24 ± 1
10.5	0.416 ± 0.002	0.042 ± 0.006	0.879 ± 0.001	112 ± 7	304 ± 11	23 ± 1
11.0	0.370 ± 0.001	0.129 ± 0.023	0.818 ± 0.009	114 ± 3	398 ± 21	35 ± 1

**Fig. 2:**
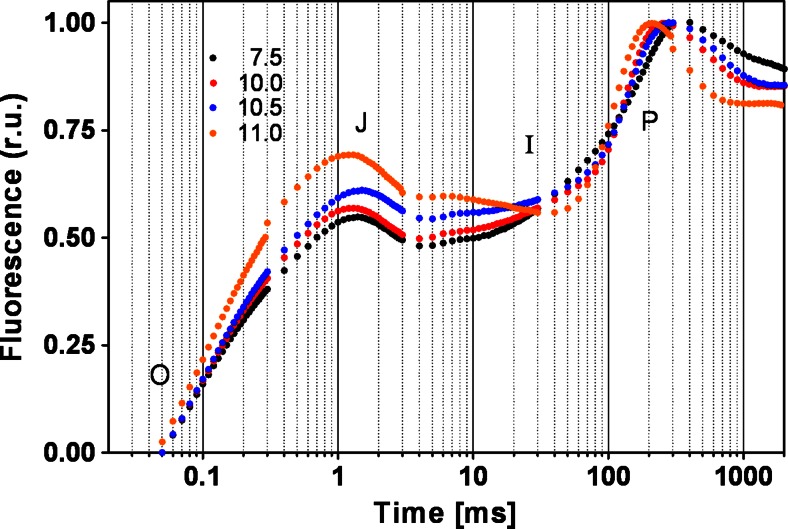
Effect of the pH on chlorophyll a fluorescence transients of the cells. Transients were normalized in both maximum and initial fluorescence values

Chlorophyll fluorescence induction kinetics (OJIP) were measured in every experiment. The transients followed the typical polyphasic OJIP rise (Fig. 2). At pH values from 7.5 to 10.0, all OJIP parameters remained stable at physiological values. At pH above 10, *M*_0_ and *V*_J_ values increased, indicating a higher rate of closure of the reaction centers and an increment in the net rate of *Q*_A_ reduction. The values of *Ψ*_0_ and *Φ*_E0_ scarcely changed within pH = 10, while they decreased by 25 and 35 % at pH = 11 respectively, evidencing a reduction in PSII’s efficiency (Supplementary Material Table S1).

*Synechocystis* cells grown at pH above 10 showed an increased optical chlorophyll cross section of about 10 % compared to cells grown at pH 7.5 (Table 2). Photosynthetic activity measured as oxygen evolution under saturating red light remained stable between pH 7.5 and 10.5 with a mean value of 335 μmol O_2_ mg chl^−1^ h^−1^, while a sharp increase (by 20 %) was observed at pH 11 (398 μmol O_2_ mg chl^−1^ h^−1^). A similar behavior was observed for dark respiration (oxygen uptake) which scarcely varied between pH 7.5 and 10.5 and increased remarkably at pH 11.

### Biochemical biomass characterization

Elemental composition (% DW) of dry biomass of *Synechocystis*, sampled at the steady state of each experiment, is reported in Supplementary Material Table S2. Carbon, nitrogen, and hydrogen contents decreased by approximately 9 % between the lowest and the highest pH. Sulfur content remained stable, except in the culture grown at pH 11 in which it was found to be 14 % lower compared to that at pH 7.5. Oxygen remained substantially stable around a value of 22 % of the DW. According to the elemental composition of *Synechocystis* biomass, the molecular mass of a C-mol was 21.83 g mol^−1^ at pH 7.5 and 22.51 g mol^−1^ at pH 11. Calcium and magnesium contents were stable at pH 7.5 and 10.0 while sodium increased by 32 %. At pH 11, sodium, calcium, and magnesium contents increased by 6.7×, 6.0×, and 2.8× times respectively (Supplementary Material Table S2).

Lipid content was stable at 12 % of DW at pH values between 7.5 and 10.0; at pH greater than 10.0, it decreased to 9.0 % (Table 3). Total protein content remained fairly constant with an average of 65.8 ± 0.5 % of DW. The amino acid profile of cells grown at three pH values (7.5, 10.0, and 11.0) did not show relevant changes (Supplementary Material Table S3). The most abundant amino acids were asparagine (mean 12.25 %) and glutamine (mean 12.94 %). Carbohydrate content increased as the alkalinity of the medium increased (Table 3). The average ash content of the biomass, at pH values between 7.5 and 10.5, was 7.0 ± 0.1 %, while at pH 11, it increased to 11.0 ± 0.3 %. DNA content increased by 14 % between pH 7.5 and 10, while a sharp increase (by 73 %) was observed at pH 11 (Table 3).

**Table 3:** Lipid, carbohydrate, protein, DNA, phycocyanin (Pc), allophycocyanin (Apc), and chlorophyll contents of *Synechocystis* cells cultured at different pH values. Values are mean ± standard deviations calculated during the steady state at each pH condition. (–) not determined

pH	Lipid (%)	Carbohydrate (%)	Protein (%)	DNA (%)	Pc (%)	Apc (%)	Chlorophyll (%)
7.5	12.1 ± 1.0	13.5 ± 0.4	66.0 ± 2.5	0.227 ± 0.036	18.6 ± 0.6	3.76 ± 0.04	2.57 ± 0.01
8.5	11.8 ± 0.3	12.1 ± 0.2	65.4 ± 1.3	–	18.2 ± 0.3	3.98 ± 0.01	2.12 ± 0.02
9.5	11.7 ± 1.4	12.7 ± 0.5	66.3 ± 3.5	–	19.5 ± 0.4	3.23 ± 0.20	2.54 ± 0.01
10.0	12.5 ± 0.1	13.6 ± 0.1	65.1 ± 0.4	0.259 ± 0.046	19.7 ± 0.3	2.07 ± 0.34	2.24 ± 0.01
10.5	9.7 ± 1.3	14.0 ± 0.6	66.4 ± 0.7	–	18.7 ± 0.5	1.69 ± 0.22	2.20 ± 0.07
11.0	9.0 ± 0.1	18.8 ± 0.4	65.5 ± 1.1	0.393 ± 0.073	13.3 ± 0.1	2.71 ± 0.14	2.00 ± 0.01

**Fig. 3:**
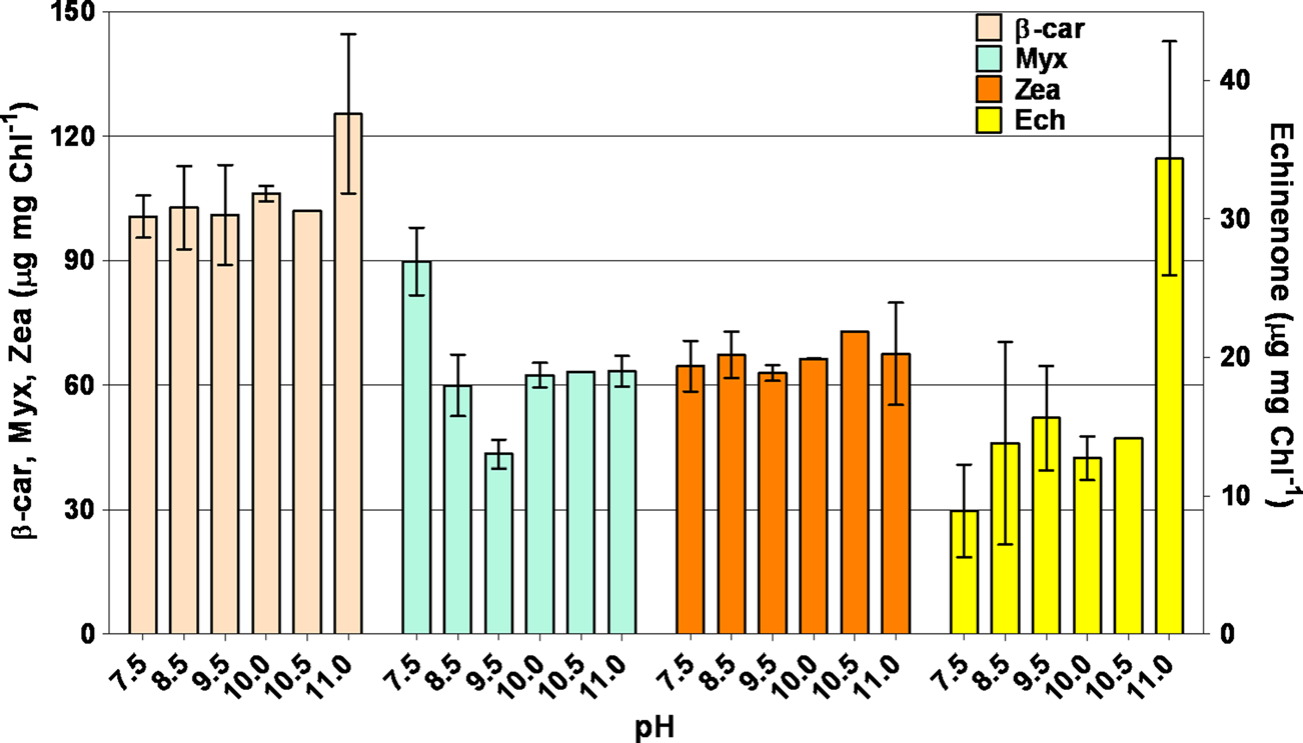
Changes in β-carotene (*β-Car*), myxoxanthophyll (*Myx*), zeaxanthin (*Zea*), and echinenone (*Ech*) detected over the various pH conditions

Pc and Apc contents decreased as pH increased (Table 3). The main carotenoids found in *Synechocystis* cultures were β-carotene (β-Car), myxoxanthophyll (Myx), zeaxanthin (Zea), and echinenone (Ech). At pH 11, increased Ech and β-Car contents were observed (Fig. 3). Chlorophyll *α* content remained at 2.0 to 2.5 % of DW.

Effect of high pH on contamination level by *Poterioochromonas* sp.

**Fig. 4:**
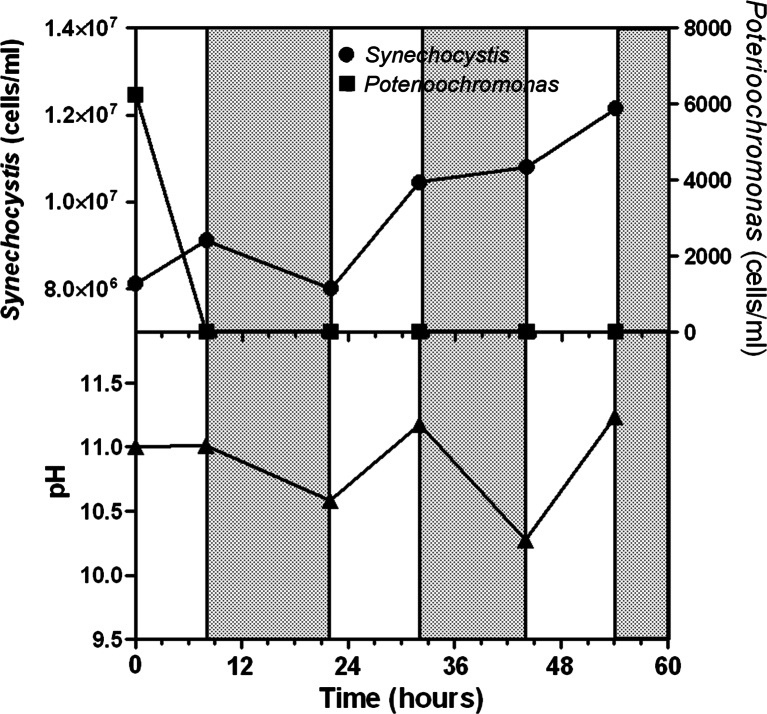
Effect of pH on *Synechocystis* and *Poterioochromonas* cell number following a sudden rise of the pH till 11.0 achieved with addition of NaOH (0.5 M) (light (*white bars*) and dark (*gray bars*) phases)

Two kinetics of pH rise were compared. For this purpose, two *Synechocystis* cultures (7.5–8.1 × 10^6^ cells mL^−1^) were contaminated with *Poterioochromonas* (initial cell concentration 6.5–7.5 × 10^3^ cells mL^−1^). In one culture, the pH was rapidly brought to 11 by adding NaOH, while in the other, the pH was left to increase as a result of the photosynthetic growth. In both cultures, pH was not allowed to exceed 11, by automatically adding CO_2_. Microscopic analysis of the culture where pH was rapidly taken to 11 revealed that after 2 h of exposure, *Poterioochromonas* cells had completely lost their motility or were spinning on themselves, and at the end of the first light period, no *Poterioochromonas* cells were detected, while *Synechocystis*’ cell number increased at a rate of 0.0145 h^−1^ (Fig. 4). However, at the end of the first dark period, *Synechocystis*’ cell number returned approximatively to the initial value, due to a remarkably high cell mortality. During the following light period, *Synechocystis*’ cell number increased, reaching more than 12 million mL^−1^. *Poterioochromonas* cells were not detected, neither in the light nor in the dark phase (Fig. 4).

**Fig. 5:**
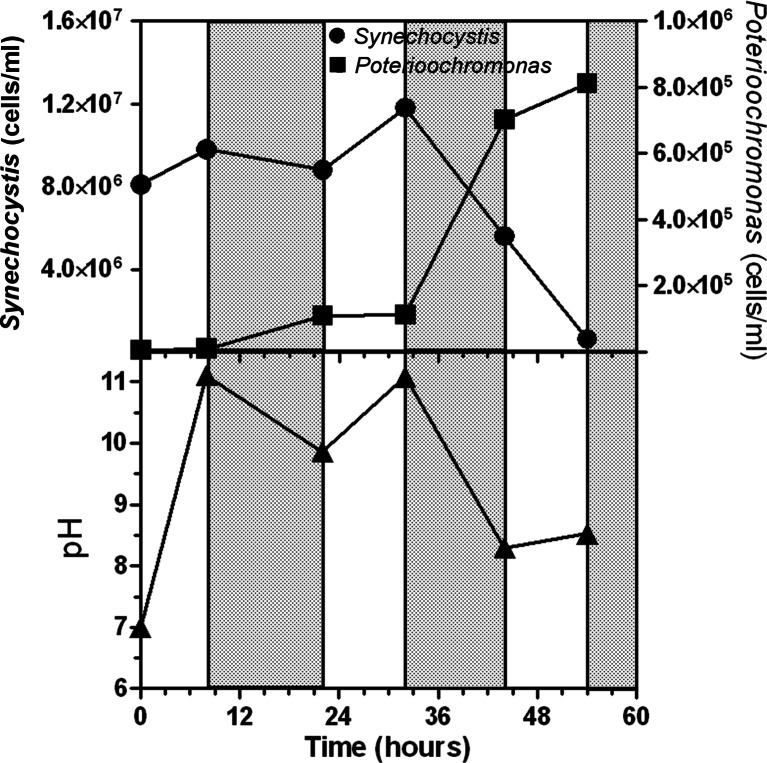
Effect of pH on *Synechocystis* and *Poterioochromonas* cell number when culture pH was allowed to increase to 11 physiologically by temporarily stopping the pH control (light (*white bars*) and dark (*gray bars*) phases)

A different behavior was observed where the pH increase to 11 was achieved physiologically, that is, along with the photosynthetic growth (CO_2_ consumption) (Fig. 5). Under such conditions, after 54 h, *Synechocystis* almost entirely disappeared and the color of the culture turned yellowish, as a result of the predominance of *Poterioochromonas* cells. The pH of the culture during the dark period usually dropped below 11, particularly at the end of the second dark period during which the pH value declined to 8.3. *Poterioochromonas* benefited of the drop of pH, becoming more motile and actively grazing *Synechocystis*. Indeed, at the end of the second dark period, their number increased almost sevenfold, outnumbering *Synechocystis* cells (Fig. 5).

**Fig. 6:**
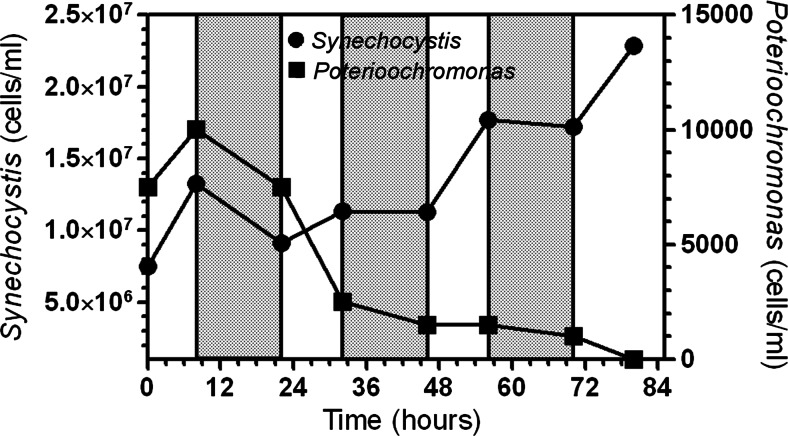
Effect of pH on *Synechocystis* and *Poterioochromonas* cell number when culture pH was constantly maintained at 11 by adding either CO_2_ (light phase (*white bars*)) or 0.5 M NaOH (dark phase (*gray bars*))

The role played by the pH during the dark phase of the cycle was studied in more detail with another experiment in which it was constantly maintained close to 11 by automatic control over the entire light-dark cycle. To minimize stress to the cells, at the start of the culture, the pH was allowed to reach 11 by growth and thereafter controlled by adding either NaOH (during the dark) or CO_2_ (during the light) (Fig. 6). Preventing the drop of pH during the dark resulted very deleterious for the survival of *Poterioochromonas* cells which, after an initial increase during which the pH was slowly rising to 11 (first light period), started to decrease in number during both the light and the dark periods (Fig. 6). *Synechocystis* cell number remained stable at about 12 million mL^−1^ in the first 48 h from the start, and increased up to 23 million mL^−1^ at the end of the experiment (80 h).

## Discussion

We found that outdoor cultures of *Synechocystis*, although grown in a closed photobioreactor, were systematically subjected to severe contamination by *Poterioochromonas* sp. (ca. 8 μm) which usually led to their complete loss within 1 week. In fact, as early as 2 days after the inoculation of the PBR outdoors, microscope observation of culture samples revealed the presence of the flagellate microalgae ingesting *Synechocystis* cells. After 6–7 days, *Synechocystis* cells had almost entirely disappeared except for sparse cell agglomerates, visible even without the aid of a microscope, while *Poterioochromonas* cells were blooming; at this stage, the cultures had completely turned to a yellowish color. It was observed that a rise of pH of the culture close to 11 in the initial stage of the contamination (first 2–3 days from inoculum) resulted in complete disappearance of the flagellate and protozoa within less than 24 h. However, to eradicate *Poterioochromonas* it was necessary to keep the pH control active and close to 11 also at night when the pH usually tends to drop as result of the respiration activity. Therefore, in view of a successful mass cultivation of *Synechocystis*, we considered of interest to focus on studying the acclimation process of this organism to high alkaline pH and the effect of such high pH on a culture’s productivity. All the pH experiments were carried out in a fixed dilution of 0.036 h^−1^, which in previous experiments proved to be optimal for high light conversion efficiency (Touloupakis et al. 2015). At this dilution rate, the resulting cell concentration of the culture with an optical path of 5 cm enabled the culture to absorb almost 100 % of the incident light, which is a condition for optimal productivity outdoors.

*Synechocystis* cultures grown close to neutral pH showed optimal productivity. Between pH 7.5 and 10, the loss in productivity was negligible (about 8 %), but increased to 32 % at pH 11. At pH 11, the light conversion efficiency was reduced to 8.9 % and a significant amount of the incident light (about 7 %) was transmitted by the culture due to the decrease in cell concentration. However, it must be pointed out that since the pH control of the cultures was achieved by adding CO_2_, and being it a substrate for photosynthesis, the amount of supplied CO_2_ directly affected photosynthesis rates and productivity. At pH 7, more CO_2_ is available for growth. However, there are evidences that when the pH of the culture is adjusted by adding a buffer, the optimal pH for growth was higher than 7.5, i.e., close to 10 (Eaton-Rye et al. 2003; Kurian et al. 2006). It has been suggested that at pH 10, an increased coupling of the phycobilisomes to PSII occurs, together with an increased transcript abundance of oxidative stress-responsive genes enhancing resistance of PSII to oxidative stress (Summerfield et al. 2013). Inorganic carbon availability, therefore, is a key factor to consider when setting up a cyanobacterial cultivation. The main forms of dissolved inorganic carbon (DIC) are carbon dioxide, bicarbonate, and carbonate. The equilibrium concentrations between these three species are pH dependent. At pH 10, the amount of CO_2_ is zero, while bicarbonate species prevail (68 % of total DIC), and the rest is represented by CO_3_^2−^ which is not utilizable by the cells. A further increase of pH to 11 reduces the HCO_3_^−^ availability to 17.6 %. The pH 11 results incompatible for the growth of most of contaminants (ciliates, amoeba, rotifers) and for the survival of *Poterioochromonas*. At pH 11, *Synechocystis* cells have still the ability to utilize HCO_3_^−^ as carbon source, provided the presence of sufficient amount of Na^+^ utilizing the Na^+^-dependent HCO_3_^−^ symporter (So et al. 1998; Badger and Price 2003), which may account for the strong accumulation of Na (6.7-fold) found at pH 11, compared to that at pH 7.5. Moreover, *Synechocystis* can rely on a CO_2_-concentrating mechanism (CCM) which affords intracellular concentrations up to three orders of magnitudes higher than those in the external medium (Kaplan and Reinhold 1999; Giordano et al. 2005). These capabilities allow the organism to cope with harsh environmental conditions without remarkable loss in productivity. On the contrary, *Synurophyceae* (heterokont) algae, such as *Poterioochromonas* sp., lack CCMs (Giordano et al. 2005, Ball 2003), and there are evidences that their phagotrophy is light dependent, although only 7 % of their total carbon budget derives from photosynthesis, indicating that there is a necessity for some factor(s) synthesized during autotrophic growth (Caron et al. 1993; Zhang and Watanabe 2001). Moreover, this organism resulted very sensitive to pH shock, resulting in a rapid loss of its motility followed by cell lysis within a couple of hours when the pH was suddenly increased to 11.

The major carotenoids found in *Synechocystis* cells at the various pH values were β-Car, Myx, Zea, and Ech. At pH 11, a higher Ech amount was found. It has been reported that in *Synechocystis*, Ech can establish a high-affinity bond with the orange carotenoid protein, activating the photoprotection mechanism (Kirilovsky 2007).

*Synechocystis*’ PSII activity, measured as *F*_v_/*F*_m_ and oxygen evolution rates, resulted unaffected by high pH values in the medium. This is in accordance with the study by Summerfield and Sherman where they observed no changes in PSII abundance between neutral and alkaline conditions (Summerfield and Sherman 2008). At pH 11, the rate of photosynthesis was stimulated by about 23 % with respect to pH 7.5, most likely as a result of acclimation of cells to higher light. Indeed, at pH 11, cell concentration (dry weight) decreased, allowing a higher light availability. Moreover, at pH 11, cells need to cope with a higher demand of ATP required by the HCO_3_^−^/Na^+^ symport (Giordano et al. 2005) and by increased maintenance energy (Touloupakis et al. 2015). At very alkaline pH, carbonate mineralization by cyanobacteria is favored, thus forming an external surface layer (S-layer) of cell wall (Kanennaya et al. 2012; Markou and Georgakakis 2011). The increase of sodium, calcium, and magnesium content, at pH 11, supports the increase by 57 % of the ash content.

In conclusion, we report evidence that, in case of contamination by *Poterioochromonas* sp., an outdoor culture of *Synechocystis* is still viable if pH is maintained above 11, which allows the complete arrest of such contaminants with an acceptable loss in productivity and no alteration in the biochemical composition of the biomass. This strategy was validated by growing cultures in a large outdoor photobioreactor (1300 L). Simply switching off the pH control unit, the culture’s pH naturally rose to values close to 11 during the day, as a result of CO_2_ uptake during photosynthesis. Night respiration entailed a reduction of pH to 9.0–9.5, which must be prevented by keeping pH at 11 by automatic addition of NaOH solution.

## Electronic supplementary material

ESM 1 (PDF 224 kb)
