# Supplementary material for: Effect of high pH on growth of Synechocystis sp. PCC 6803 cultures and their contamination by golden algae (Poterioochromonas sp.)
Source: Appl Microbiol Biotechnol. 2015 Nov 6;100:1333–41. doi: 10.1007/s00253-015-7024-0 (PMC4717179; doi:10.1007/s00253-015-7024-0)
Supplement: Supplementary file 1 — (PDF 224 kb) [file 253_2015_7024_MOESM1_ESM.pdf]

Supplemental Materials

Effect of high pH on growth of *Synechocystis* sp. PCC 6803 cultures and their contamination by golden algae (*Poterioochromonas* sp.)

Applied Microbiology and Biotechnology

Eleftherios Touloupakis[1], Bernardo Cicchi[1], Ana Margarita Silva Benavides[2,3], Giuseppe Torzillo[1]*

[1]Istituto per lo Studio degli Ecosistemi, CNR, Via Madonna del Piano, 10, I-50019 Sesto Fiorentino, Italy.
[2]Escuela de Biología, [3]Centro de Investigación en Ciencias del Mar y Limnología (CIMAR), Universidad de Costa Rica, San Pedro, San José 11501, Costa Rica.

*corresponding author: Tel.: +39 055 5225992; Fax: +39 055 5225920, E-mail: torzillo@ise.cnr.it

Figure S1. Light microscopy of *Synechocystis* PCC 6803 cells. A) axenic culture; B) contaminated by *Poterioochromonas* sp. which usually after five days almost completely grazed *Synechocystis* cells. Dashed arrows indicate free Synechocystis cells; filled arrows show ingested *Synechocystis* cells.

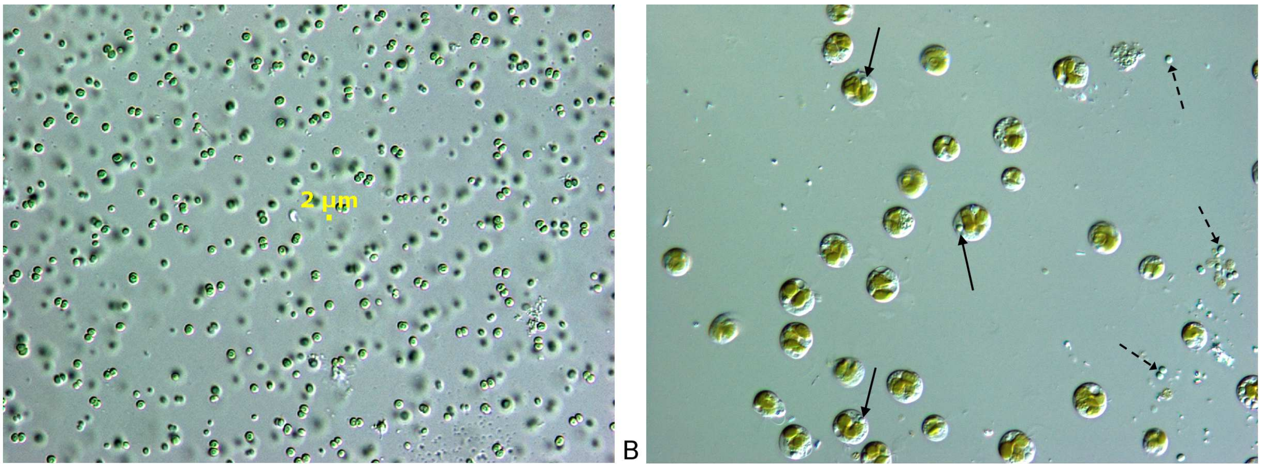

A B

Table S1. The M_0 (initial slope at the beginning of the variable fluorescence), V_J (the variable fluorescence at phase J), Φ_{E0} (the quantum yield of electron transport), Ψ_0 (the efficiency with which a trapped exciton can move an electron further than Q_A^- into the electron transport chain) and the parameter Sm of *Synechocystis* cultured at different pH conditions. Values are mean ± standard deviations calculated over the steady state for each pH condition.

pH	M_0	V_J	Ψ_0	Φ_{E0}	S_m
7.5	1.47±0.06	0.514±0.025	0.486±0.025	0.165±0.007	59.0±3.7
8.5	1.60±0.02	0.543±0.004	0.456±0.004	0.138±0.002	58.0±0.8
9.5	1.57±0.03	0.543±0.010	0.457±0.010	0.146±0.002	59.1±1.1
10.0	1.63±0.02	0.543±0.010	0.457±0.010	0.150±0.003	53.4±2.0
10.5	1.69±0.04	0.594±0.013	0.406±0.013	0.135±0.006	53.7±1.4
11.0	2.02±0.06	0.662±0.018	0.344±0.008	0.097±0.005	49.6±0.8

Table S2. Elemental composition of *Synechocystis* cells cultured at different pH conditions. Values are mean ± standard deviations calculated during the steady state at each pH condition. (-) not determined.

pH	C (%)	N (%)	H (%)	S (%)	O (%)	Na (%)	Ca (%)	Mg (%)
7.5	48.0±0.1	11.3±0.3	6.88±0.17	0.418±0.014	21.1±0.4	0.118±0.001	0.270±0.008	0.364±0.006
8.5	46.1±0.4	11.5±0.6	6.63±0.01	0.425±0.017	21.8±1.1	-	-	-
9.5	44.7±0.2	10.3±0.1	6.64±0.07	0.411±0.010	21.9±0.7	-	-	-
10.0	45.1±0.2	10.3±0.2	6.72±0.08	0.411±0.016	22.5±0.6	0.079±0.001	0.285±0.004	0.367±0.008
10.5	44.9±0.3	10.2±0.1	6.77±0.20	0.423±0.013	23.2±0.2	-	-	-
11.0	43.8±2.5	10.3±0.3	6.26±0.30	0.362±0.039	21.9±0.4	0.789±0.010	1.660±0.021	1.028±0.017

Table S3. Amino acid composition of *Synechocystis* cultures grown at three different pH conditions.

Amino acid % (w/w)	pH 7.5	pH 10.0	pH 11.0
**His**	1.41±0.01	1.52±0.00	1.39±0.01
**Ser**	3.81±0.01	3.53±0.02	3.85±0.01
**Arg**	9.99±0.01	10.01±0.01	9.57±0.01
**Gly**	4.50±0.01	5.35±0.00	4.58±0.01
**Asp**	12.68±0.02	11.33±0.01	12.75±0.01
**Glu**	13.12±0.01	11.97±0.01	13.72±0.01
**Thr**	4.98±0.01	4.65±0.01	5.15±0.01
**Ala**	9.24±0.02	8.82±0.01	9.03±0.02
**Pro**	3.79±0.01	5.01±0.00	3.70±0.01
**Cys**	0.09±0.00	0.08±0.00	0.10±0.00
**Lys**	5.49±0.01	4.98±0.01	6.39±0.01
**Tyr**	2.74±0.05	3.32±0.01	2.00±0.00
**Met**	1.71±0.01	1.74±0.00	1.52±0.01
**Val**	6.29±0.01	6.37±0.01	6.40±0.01
**Ile**	5.97±0.03	6.14±0.00	6.03±0.01
**Leu**	9.75±0.01	10.03±0.01	9.67±0.01
**Phe**	4.42±0.06	5.13±0.01	4.11±0.00
